# A Third *MLPH* Variant Causing Coat Color Dilution in Dogs

**DOI:** 10.3390/genes11060639

**Published:** 2020-06-10

**Authors:** Samantha L. Van Buren, Katie M. Minor, Robert A. Grahn, James R. Mickelson, Jennifer C. Grahn, Julia Malvick, Jennifer R. Colangelo, Elisabeth Mueller, Petra Kuehnlein, Alexandra Kehl

**Affiliations:** 1 Department of Veterinary Clinical Sciences, University of Minnesota, St. Paul, MN 55108, USA; vanbu040@umn.edu; 2 Department of Veterinary and Biomedical Sciences, University of Minnesota, St. Paul, MN 55108, USA; micke001@umn.edu; 3 Veterinary Genetics Laboratory, School of Veterinary Medicine, University of California at Davis, Davis, CA 95616, USA; ragrahn@ucdavis.edu (R.A.G.); jcgrahn@ucdavis.edu (J.C.G.); jzcollins@ucdavis.edu (J.M.); jrcolangelo@ucdavis.edu (J.R.C.); 4 Laboklin GmbH&Co.KG, Steubenstraße 4, D-97688 Bad Kissingen, Germany; mueller@laboklin.de (E.M.); Kuehnlein@laboklin.de (P.K.); kehl@laboklin.com (A.K.)

**Keywords:** *Canis lupus*, mammalian pigmentation, melanophilin, coat color dilution, coat color genes, coat color phenotypes

## Abstract

Altered melanosome transport in melanocytes, resulting from variants in the melanophilin (*MLPH*) gene, are associated with inherited forms of coat color dilution in many species. In dogs, the *MLPH* gene corresponds to the D locus and two variants, c.−22G > A (d^1^) and c.705G > C (d^2^), leading to the dilution of coat color, as described. Here, we describe the independent investigations of dogs whose coat color dilution could not be explained by known variants, and who report a third *MLPH* variant, (c.667_668insC) (d^3^), which leads to a frameshift and premature stop codon (p.His223Profs*41). The d^3^ allele is found at low frequency in multiple dog breeds, as well as in wolves, wolf-dog hybrids, and indigenous dogs. Canids in which the d^3^ allele contributed to the grey (dilute) phenotype were d^1^/d^3^ compound heterozygotes or d^3^ homozygotes, and all non-dilute related dogs had one or two D alleles, consistent with a recessive inheritance. Similar to other loci responsible for coat colors in dogs, this, alongside likely additional allelic heterogeneity at the D locus, or other loci, must be considered when performing and interpreting genetic testing.

## 1. Introduction

Natural and artificial selection in dogs has led to a myriad of phenotypes with respect to coat coloration and its patterns. Two pigments, phaeomelanin (red/yellow) and eumelanin (black/brown), in which physical changes to the pigments themselves, as well as the timing and location of their deposition, contribute to a variety of color phenotypes. One such phenotype, dilution, is found in multiple mammalian and avian species. In dogs, the D (dilution) locus causes lightening of the coat color—i.e., from black to grey, brown to silver, or red to cream—as well as pigmentation lightening of the nose, paw pads, and eye color. 

Melanosome transport, along cortical actin filaments in melanocytes, is regulated by three major proteins: Rab27A (a member of the small GTPase Rab family), the motor protein myosin Va (MYO5A), and melanophilin (*MLPH*) [1]. These three proteins form a functional ternary complex, in which an alteration in any of them can result in defects in melanosome transport and a dilute phenotype [1]. In 2007, Drögemüller et al. identified a transition at the splice boundary of the first untranslated exon of the canine *MLPH* gene, predicted to result in reduced splicing efficiency [2]. This non-coding variant, c.−22G > A (d^1^), was associated with the dilution phenotype in seven breeds of dogs at the time [2]. A small percentage of dilute dogs did not possess a d^1^ genotype predicted to result in the dilution phenotype, and Bauer et al. (2018) subsequently identified a second *MLPH* variant, c.705G > C (d^2^), located at the splice boundary of exon 7, and correlating to dilution in three additional breeds [3]. 

The effects of *MLPH* variants on melanosome transport and pigmentation are distinct from the recently identified variants of phaeomelanin-based pigment intensity, which also perturb the perceived depth of coloration, although resulting coat colors are varying shades of red, cream, and white [4]. Nevertheless, the previously identified d^1^ and d^2^ *MLPH* variants do not explain all of the dilute coat color phenotypes in canids, and a recent report suggests that additional coat color alleles are likely present at low frequency throughout the canine population [2,3,5]. Here we combined candidate gene sequencing with a query based on a large dataset of whole genome sequences, to identify a novel *MLPH* variant (d^3^), associated with dilute coat color in multiple dog breeds and wolf-dog hybrids.

## 2. Materials and Methods 

### 2.1. Animals

Cheek swab DNA samples were collected from 16 dilute dogs (3 Hungarian pumis, 5 Hungarian mudis, 5 wolf-dog hybrids, 1 Chihuahua, 1 Italian greyhound, and 1 Pekingese) that were previously genotyped for the d^1^ and d^2^ *MLPH* alleles, and incorrectly predicted to have a non-diluted coat color and 1 diluted d^1^/d^1^ Shih Tzu, along with 32 non-dilute dogs (11 Hungarian pumis, 15 Hungarian mudis, 2 wolf-dog hybrids, 1 Pekingese, and 3 Shih Tzu). Written consent was obtained from all dog owners. This study was approved under University of Minnesota IACUC protocol 1903 36865A. 

### 2.2. MLPH Exon Sequencing

Genomic DNA was extracted from the cheek swab samples using Qiagen’s Puregene tissue kit or Qiagen’s DNEasy DNA extraction kit. The entire coding sequence (except exon 11), as well as flanking intronic boundaries, were Sanger sequenced in the Italian greyhound (Supplementary Table S1). For the pumis, mudis, Chihuahua and wolf-dog hybrids, an amplicon encompassing exon 7 of the *MLPH* gene, and extending into the flanking introns (including alleles d^2^ and the 1 bp insertion reported here), was obtained (Supplementary Table S1). For the mudis and wolf-dog hybrids not previously genotyped for the d^1^ allele, a 268 bp amplicon was obtained with d^1^ PCR primers F and R (Supplementary Table S1). Genotyping was performed with direct Sanger sequencing of the amplicon at Eurofins Genomics (Louisville, KY, USA), and visualized with Sequencher software (Gene Codes Corporation, Ann Arbor, MI, USA), or sequenced by Laboklin and analyzed with SeqScanner Software, respectively. 

### 2.3. Whole-Genome Database Query

The coding sequence and surrounding splice site regions of the canine *MLPH* gene, were inspected for potential loss-of-function mutations in a variant call file (VCF). This contained 804 dog (from >140 breeds) and 9 wolf genomes, available in an updated version of the Dog Biomedical Variant Database Consortium (DBVDC), utilizing Golden Helix GenomeBrowse, with public annotations from Ensembl Genes 89 and RefSeq Genes 104 [6,7].

## 3. Results

### 3.1. MLPH Exon Sequencing

**Figure 1:**
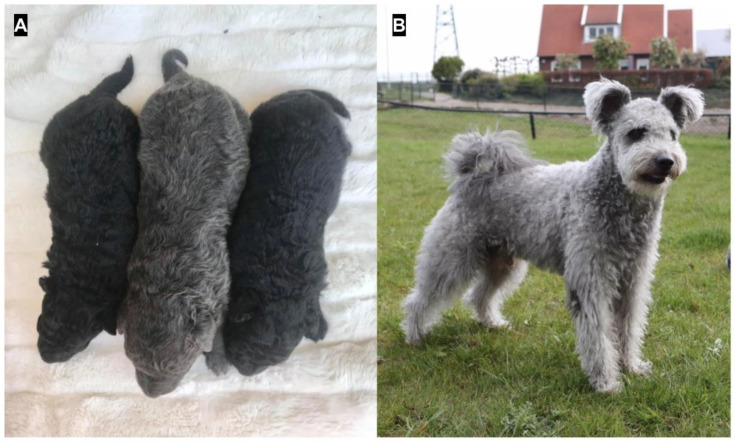
Examples of dogs with a dilute phenotype. **A**, a dilute Hungarian mudi puppy flanked by its black siblings; **B**, a dilute Hungarian pumi.

Examples of dogs with dilute phenotypes included in our study are provided in Figure 1. Our initial investigations focused on the Hungarian pumi and mudi dogs, as well as an Italian greyhound, whose dilute phenotypes were not explained by the d^1^ and d^2^ variants genotyped by a commercial laboratory; i.e., these dilute dogs were not homozygous for the d^1^ (c.−22G > A) or d^2^ (c.705G > C) variants, and they were not compound heterozygotes (d^1^/d^2^). The *MLPH* coding sequence, as well as flanking intronic boundaries, were sequenced in the Italian greyhound, and exons 1 and 7 were sequenced in the pumis and mudis. We confirmed the commercial laboratory results, where the dilute Italian greyhound and dilute pumis were heterozygous for d^1^ and homozygous wild type for d^2^. At the same time, we identified a cytosine insertion subsequent to a run of 8 cytosine bases, (NM_001103219.2: c.667_668insC or chr25: g.48150749_50insC (CanFam 3.1 assembly), hereafter referred to as d^3^. No other variants altering the protein coding sequence were found.

**Figure 2:**
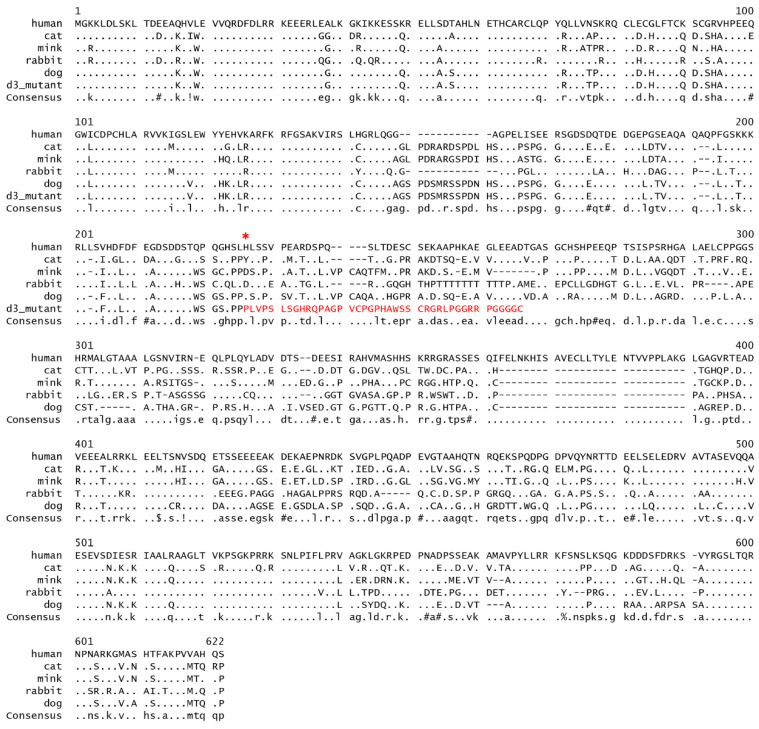
Amino acid sequence alignment of mammalian *MLPH* genes. The transcript IDs for the alignments are as follows: Human, NM_024101.7; cat, ENSFCAT00000039067.3; American mink, ENSNVIT00000032578.1; rabbit, ENSOCUT00000016497.4; and dog, NM_001103219.2. The position of the d^3^ dog variant is indicated with an *, and the recoded peptide preceding the premature stop codon follows in **red**.

The canine *MLPH* gene contains 16 exons and 581 amino acid residues. As expected, considerable cross species homology exists across the full length of the protein; however, several regions of poor conservation are also present (Figure 2). The (c.667_668insC, p.His223Profs*41) insertion is predicted to recode 41 amino acids, before a stop codon is generated that truncates 54% of the wild type protein. 

### 3.2. Variant Database Search

Inspection of the DBVDC VCF containing 804 dog and 9 wolf genomes, also revealed the previously unrecognized 1 bp insertion (d^3^) within exon 7, at an allele frequency of 0.0045. The d^3^ variant was present in 7 genomes in the heterozygous state (4 indigenous dogs, 1 Tibetan mastiff, 1 Yorkshire terrier, and 1 wolf), and no homozygous d^3^ individuals were found. Additionally, 1 heterozygous Shetland sheepdog was identified within the University of Minnesota’s private database. The frequencies of the d^1^ and d^2^ alleles in the DBVDC are 0.054 and 0.0025, respectively.

### 3.3. MLPH Genotyping

**Figure 3:**
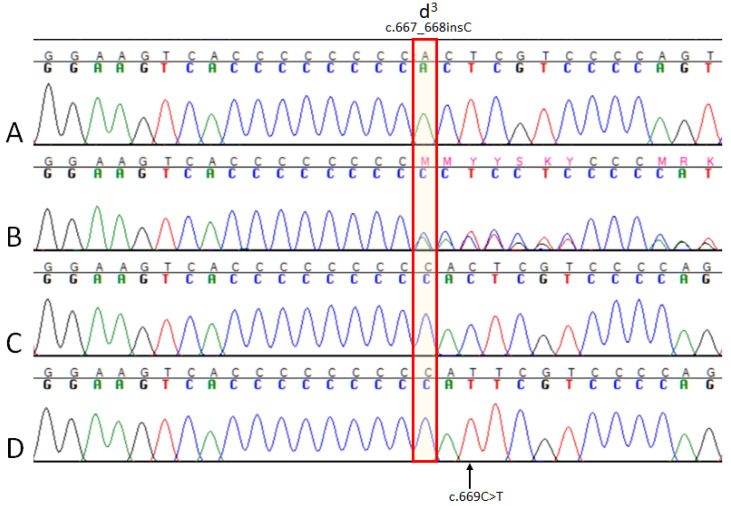
Sanger sequencing traces from *MLPH* exon 7. The **red** box highlights the location of the d^3^ variant. **A**, a D/D mudi; **B**, a D/d^3^ mudi; **C**, a d^3^/d^3^ mudi; **D**, a d^3^/ d^3^ wolf-dog hybrid that also has a synonymous SNP (c.669C > T) 2 bp from the d^3^ mutation.

Sanger sequencing enabled us to resolve all three d^3^ variant genotypes, with the d^3^ variant homozygous in the dilute mudi (Figure 3). In addition to the d^3^ variant in the dilute wolf-dog hybrid, a previously recognized synonymous variant, c.669C > T (dbSNP rs852237859), was also observed. This SNP is relatively common in the DBVDC dogs at an allele frequency of 0.062. None of the dogs with the d^3^ allele in DBVDC have this SNP, while the wolf from DBVDC with the d^3^ allele does. The d^2^ variant (also in exon 7) was not observed in our Sanger-sequenced study cohort.

### 3.4. Genotype-Phenotype Association

Table 1 and Supplementary Table S2 present the *MLPH* variant genotypes from the available phenotyped dogs, including 14 Hungarian pumis, 20 Hungarian mudis, 7 wolf-dog hybrids, 1 Chihuahua, 1 Italian greyhound, 2 Pekingese, and 4 Shih Tzu. 

A perfect association between the d^3^ variant and the dilute coat color was found in three pumi families: all three grey pumis were compound heterozygotes (d^1^/d^3^), whereas all non-dilute pumis had at least one D allele. The three families—P1–4, P5–11 and P12–14—were also investigated for allelic segregation within the phenotype. No sire was available for families P1–4 and P12–14. The available female P1 (mother of P2–P4) and P12 (mother of P13 and P14) carried one d^1^ allele, which was transmitted to one of their respective offspring. Both pups were phenotypically grey and had d^1^/d^3^ genotypes, and thus inherited the d^3^ allele form the sire. In the third family (P5–P11), both parents were heterozygotes: the mother for d^3^ and the father for d^1^. Four descendants showed a black coat color being homozygous D/D or heterozygous D/d^1^ and D/d^3^, and one pup was compound heterozygous d^1^/d^3^ and showed a grey coat. 

The D locus genotypes for the 20 phenotyped mudi are also shown in Table 1. The dilute mudi were either d^1^/d^3^ compound heterozygotes or d^3^/d^3^ homozygotes, while all non-dilute dogs had at least one D allele. Transmission of the d^3^ allele was also demonstrated in a pedigree that produced both dilute (d^3^/d^3^) and non-dilute (D/d^3^) offspring. 

Similarly, the grey wolf-dog hybrids were discordant at their d^1^ locus genotype. These dogs were evaluated at the d^2^ and d^3^ loci, and the genotypes d^1^/d^3^ or d^3^ /d^3^ were confirmed, respectively. The mother and sibling of the d^1^/d^3^ heterozygote were subsequently genotyped. The d^1^ allele was inherited from the mother (AW1), and the d^3^ allele was likely inherited from the father, or represented a spontaneous mutation event. In three additional unrelated dilute wolf-dog hybrids (Figure 3), the previously recognized synonymous variant, c.669C > T (dbSNP rs852237859), was observed in addition to the d^3^ frameshift variant. The d^2^ variant was not observed in any of the sequenced samples. Additionally, the DBVDC database contained 9 wolves: 7 were wt/wt at both d^1^ and d^3^, 1 was heterozygous at d^1^ only, and 1 was heterozygous at d^3^ only.

Additional examples of the association between d^1^ and d^3^ variants, with dilute coat color in smaller cohorts of other breeds, included the Shih Tzu, Chihuahua, Pekingese, and Italian greyhound (Table 1). Examples of D/d^3^ heterozygotes (4 indigenous dogs, 1 Tibetan mastiff, 1 Yorkshire terrier, 1 wolf and 1 Shetland sheepdog) were identified in the aforementioned WGS variant databases. A single homozygous d^3^ Shih Tzu has been identified (ShT3) (Table 1, Table S2), but this individual was also homozygous for the d^1^ allele. The non-dilute offspring (ShT5 and 6) from this individual, and a male wild type at all three known variants (ShT4), were heterozygous for both the d^1^ and d^3^ alleles. However, from knowledge of the parents′ genotypes, it can be deduced that the pups received a chromosome containing both the d^1^ and d^3^ variants from the dam, and a D chromosome from the sire, so the offspring still had one functional D allele. In this case, the d^1^/d^3^ haplotype likely represents either a spontaneous mutation at the d^3^ locus on the d^1^ background that is identical by state, or a recombination event. 

**Figure 4:**
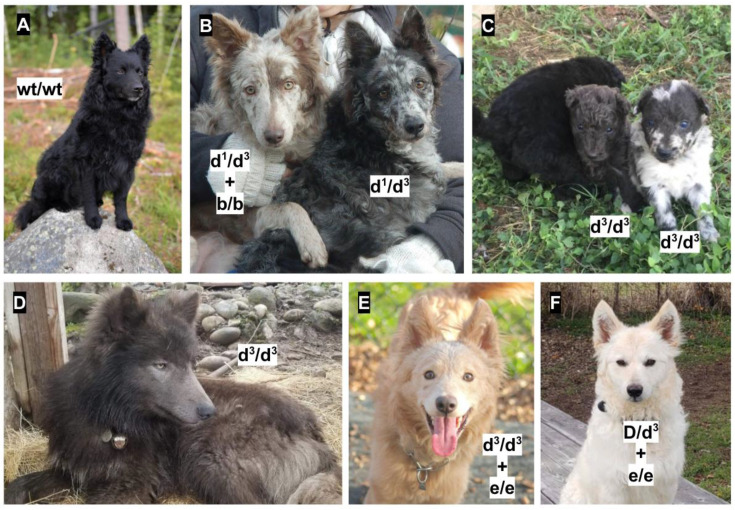
Hungarian mudi dogs and wolf-dog hybrids with a dilute coat color. **A**, a homozygous wild type mudi dog at d^1^, d^2^, and d^3^; **B**, two mudi dogs that are both compound heterozygotes for d^1^ and d^3^; **C**, two mudi dogs homozygous for d^3^; **D**, a d^3^ homozygous wolf-dog hybrid; **E**, an e/e, d^3^ homozygous mudi dog; **F**, an e/e, D/d^3^ mudi dog.

Figure 4 provides additional images of the dogs genotyped in our study, and the impacts of additional coat color loci. Both mudis in (B), the mudi on the right in (C), and the mudis in (E) and (F) also have the merle mutation [8]. The merle mutation is inherited as autosomal dominant, and often results in uneven patches of pigment diluted to light grey, light brown, or white [8,9]. However, it is not phenotypically apparent in phaeomelanin. Additionally, the dog on the left in (B) has a *TYRP1* recessive brown mutation [10,11,12]. Dogs homozygous recessive at both *TYRP1* and *MLPH*, are diluted at brown instead of black, and have a resulting pigment color referred to in many breeds as “silver”, “isabella”, or “lilac”. In mudis, this color is referred to as “grey-brown”. The mudis in (E) and (F) have two copies of the recessive e allele of the *MC1R* gene, that affects phaeomelanin and results in varying shades of cream [4,10,13,14,15,16]. Additionally, the mudi in (E) is homozygous for d^3^, thus showing grey pigmentation on the nose and eye rims, and a lightened eye color. 

## 4. Discussion

*MLPH* gene variants cause coat color dilution in dogs and other species [17,18,19,20,21]. However, not all dogs that are phenotypically dilute are explained by the two published *MLPH* dilution variants [2,3]. The d^3^ variant was identified at the UC Davis Veterinary Genetics Laboratory, in exon 7 of a phenotypically dilute Italian greyhound, with one copy of d^1^ and no copies of d^2^. At the same time, the d^3^ variant was detected at Laboklin in dilute pumis and wolf-dog hybrids, where testing for d^1^ and d^2^ confirmed the dogs to be compound heterozygous d^1^/d^3^. Independently, a scan of the DBVDC for other *MLPH* coding sequence variants by the University of Minnesota, identified the same frameshift mutation in exon 7, after being presented with dilute mudis and wolf-dog hybrids, who were tested previously as D/D or D/d^1^. All three independent identifications confirmed the mutation through Sanger sequencing. 

**Figure 5:**
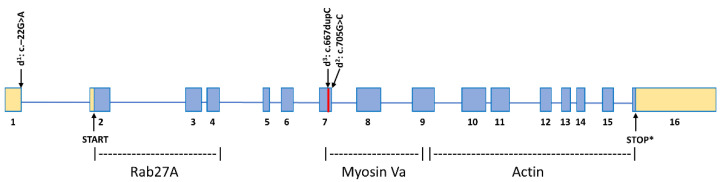
Locations of the d^1^, d^2^ and d^3^ variants within the *MLPH* gene. Exons are boxes, protein coding regions are blue and non-coding regions are tan. Protein interaction domains were adapted from a previous analysis in rabbit and American mink [18,21].

Exon 7 harbors two of the three known canine *MLPH* mutations (Figure 5). The region is not highly conserved across mammals (Figure 2); however, it is in the vicinity of a predicted myosin Va interaction site, necessary for facilitating melanosome transport. The ablation of MLPH-myosin Va interaction, therefore, readily explains both the defective melanosome transport and the dilute grey phenotype. Taken together, the above arguments strongly suggest that the variant, c.667_668insC, contributes to the dilution of coat color in multiple breeds, and represents another loss-of-function allele similar to d^1^ and d^2^. We observed the association of the d^3^ allele with dilute coat color, both in its homozygous form and as a compound d^1^/d^3^ heterozygote. That compound heterozygosity can result in the dilute coat color phenotype, has previously been shown with the d^1^ and d^2^ variants [3].

The d^3^ allele was found in a wide spectrum of breeds and other canids, minimally including the Hungarian pumi, Hungarian mudi, Chihuahua, Pekingese, Italian greyhound, Shih Tzu, Tibetan mastiff, Yorkshire terrier, and Shetland sheepdog, as well as indigenous dogs, wolves, and wolf-dog hybrids. The wolf-dog hybrids in this study that carry the d^3^ variant have recent ancestry behind them, including wolf, Alaskan Malamute, German shepherd dog, Siberian husky, and collie. The presence of the d^3^ variant in village dogs and wolves may indicate that this variant represents an ancient mutation, persisting at a low frequency across some breeds, while being lost in others. However, given that this variant is a single nucleotide insertion in an 8 bp poly C stretch, the possibility that d^3^ is identical by state, at least in some breeds, must be considered. The presence of the d^3^ variant on the d^1^ background in the Shih Tzu, and the c.669C > T variant 2 bp from the wolf d^3^ allele, would suggest that this type of spontaneous mutation can occur. Alternatively, this could represent a recombination event between the d^1^ and d^3^ haplotypes. Regardless, the identified Shih Tzu had two copies of the d^1^/d^3^ haplotype, suggesting that this specific allele is present, at least in this breed.

Despite a third dilution variant being discovered, not all phenotype/genotype discrepancies are explained. Overall, 99.74% of > 10,000 genotyped phenotypically dilute French bulldogs, as reported by their owners, are resolved by the known d^1^ and d^2^ variants, and the d^3^ variant has not yet been detected in this breed at all (private lab observation, data not shown). Since every discordant dog is heterozygous at d^1^, and homozygous wild type at both d^2^ and d^3^, it is possible that at least one additional *MLPH* variant exists, or a variant in a different gene altogether.

This novel d^3^ variant adds to our understanding of the basis for coat color variation, and the interaction between variants in dogs and other species. Additionally, known coat color variants are useful to breeders in understanding the potential outcomes of matings. As this newly identified c.667_668insC d^3^ variant does not resolve all *MLPH* genotype/phenotype inconsistencies, additional variants are likely. This fact must be taken under consideration when performing genetic testing and counseling. 

## Figures and Tables

**Table 1:** *MLPH* variant genotypes in phenotyped dogs.

Breed	Color	D/D	D/d^1^	D/d^3^	d^1^/d^3^	d^1^/d^1^	d^3^/d^3^
Hungarian pumi	Black	5	4	2	0	0	0
Hungarian pumi	Grey	0	0	0	**3**	0	0
Hungarian mudi	Black *	5	3	6	0	0	0
Hungarian mudi	Grey *	0	0	0	**2**	0	**2**
Hungarian mudi	Cream *	0	0	1	0	0	**1 #**
Wolf-dog hybrid	Black	0	1	0	0	0	0
Wolf-dog hybrid	Grey	0	0	0	**1**	**1**	**3**
Wolf-dog hybrid	Other	1	0	0	0	0	0
Chihuahua	Grey	0	0	0	**1**	0	0
Italian greyhound	Grey	0	0	0	**1**	0	0
Pekingese	White	0	0	0	**1 #**	0	0
Pekingese	Brown	0	1	0	0	0	0
Shih Tzu	White	1	0	0	0	0	0
Shih Tzu	Grey	0	0	0	0	1 **	
Shih Tzu	Non-dilute	0	2 **		0	0	0

Dogs were genotyped by Sanger sequencing as described in Methods. *, mudi with merle patterning are included; **, Shih Tzu chromosome containing both d^1^ and d^3^ variants; **#**, cream/white dogs with lightened nose, paw pad, and eye color. Genotypes capable of producing the dilute coat color are shaded in **grey**.

## Supplementary Materials

The following are available online at https://www.mdpi.com/2073-4425/11/6/639/s1, Table S1: Primers used for the PCR amplification and sequencing of *MLPH* exons, Table S2: Genotypes of all dogs in this study.
