# Supplementary material for: A Third MLPH Variant Causing Coat Color Dilution in Dogs
Source: Genes (Basel). 2020 Jun 10;11(6):639. doi: 10.3390/genes11060639 (PMC7349360; doi:10.3390/genes11060639)
Supplement: Supplementary file 1 [file genes-11-00639-s001.zip › genes-824794-supplementary.docx]

**Table S1.** Primers used for sequencing and genotyping, *A single sequenceable product was not obtained with these primers.

| **Name** | **Sequence** | **Amplicon size** |
| --- | --- | --- |
| K9_MLPH_Ex1_F | CAGAATTGGGTAGGAATCCA | 659 |
| K9_MLPH_Ex1_R | GAAAGGGAGTGCCCTCTG |  |
| K9_MLPH_Ex2_F | AGTTCCAAGTGTGCAGGAAC | 592 |
| K9_MLPH_Ex2_R | CTGAAGCTGGATCAAGAGGA |  |
| K9_MLPH_Ex3-4_F | GGAGACCCAGGATCGAGT | 728 |
| K9_MLPH_Ex3-4_R | CACACTCACATTCCAAAGGA |  |
| K9_MLPH_Ex5_F | GAAGTGATGGAGGTCACTGTT | 517 |
| K9_MLPH_Ex5_R | TGCATGAGTGTGCTTGTGTA |  |
| K9_MLPH_Ex6_F | GGGTCTAAATGATGCTTGGA | 556 |
| K9_MLPH_Ex6_R | CCTCTTTCGTGCTCAGTCTC |  |
| K9_MLPH_Ex7_F | CAGGTGTTTCCTTCTGCTCT | 580 |
| K9_MLPH_Ex7_R | CATCCCGTACACCACTTCTC |  |
| UMN_MLPH_Ex7_F | GCAGGTGTTTCCTTCTGCTC | 582 |
| UMN_MLPH_Ex7_R | CCATCCCGTACACCACTTCT |  |
| K9_MLPH_Ex8_F | GTCCCTCGTGTGATGAGTG | 685 |
| K9_MLPH_Ex8_R | ATTAGCGCTGGAACTTCTGA |  |
| K9_MLPH_Ex9_F | TAAAGTCACCTCTGCTCGTG | 617 |
| K9_MLPH_Ex9_R | TTGAAGGCTCTGATTTCTCC |  |
| K9_MLPH_Ex10_F | TCAGAGGGAAGAACTGTTGG | 528 |
| K9_MLPH_Ex10_R | CTTCGTCACAACCTCTGGAC |  |
| *K9_MLPH_Ex11_F* | *GTCCAGAGGTTGTGACGAAG* | *629 |
| *K9_MLPH_Ex11_R* | *GTCAGAGTCTGGTCCTGAGC* |  |
| K9_MLPH_Ex12_F | CCTTAGAGCTACAGCGATGC | 569 |
| K9_MLPH_Ex12_R | CCAAGTTCTCACAGGAAAGG |  |
| K9_MLPH_Ex13_F | AGGCAAACAGAGGGTGGT | 506 |
| K9_MLPH_Ex13_R | TTACCCAGAGCCAAAGACAC |  |
| K9_MLPH_Ex14_F | CTGAAGGCAGATGCTCAAC | 424 |
| K9_MLPH_Ex14_R | CTGCTTCCCTCTGGTGAC |  |
| K9_MLPH_Ex15_F | GTTGGCTAGGATGTGAGAGC | 578 |
| K9_MLPH_Ex15_R | CTTTAAATCTTTGGCCGATTT |  |
| K9_MLPH_Ex16_F | GCTTCAGAGCCTGAAATTCT | 407 |
| K9_MLPH_Ex16_R | GTACAGGAAAGCCACGTCA |  |
| d1_PCR_primer_F | CCTTCCTCCCCTGTAGGAC | 268 |
| d1_PCR_primer_R | GTAGCCCTGAGGCCTGTGT |  |

**Table S2.** D locus genotype data across breeds. Breeds are separated by double lines and families within breeds in which allele segregation can be followed are separated by a thick line.

| **Breed** | **Dog** | **Color** | **d1 Genotype** | **d2 Genotype** | **d3 Genotype** | **D locus Genotype** | **Relationship** |
| --- | --- | --- | --- | --- | --- | --- | --- |
| Pumi | P1 | greying | D/d1 | D/D | D/D | D/d1 | Dam of P2-P4 |
| Pumi | P2 | grey | D/d1 | D/D | D/d3 | d1/d3 | offspring |
| Pumi | P3 | black | D/D | D/D | D/D | D/D | offspring |
| Pumi | P4 | greying | D/D | D/D | D/D | D/D | offspring |
| Pumi | P5 | black | D/D | D/D | D/d3 | D/d3 | Dam of P7-P11 |
| Pumi | P6 | black | D/d1 | D/D | D/D | D/d1 | Sire of P7-P11 |
| Pumi | P7 | black | D/D | D/D | D/D | D/D | offspring |
| Pumi | P8 | black | D/D | D/D | D/D | D/D | offspring |
| Pumi | P9 | black | D/d1 | D/D | D/D | D/d1 | offspring |
| Pumi | P10 | grey | D/d1 | D/D | D/d3 | d1/d3 | offspring |
| Pumi | P11 | black | D/D | D/D | D/d3 | D/d3 | offspring |
| Pumi | P12 | black | D/d1 | D/D | D/D | D/d1 | Dam of P13,P14 |
| Pumi | P13 | black | D/D | D/D | D/D | D/D | offspring |
| Pumi | P14 | grey | D/d1 | D/D | D/d3 | d1/d3 | offspring |
| Mudi | Mudi02 | black | D/D | D/D | D/d3 | D/d3 | Sire of Mudi04-6,15,16 |
| Mudi | Mudi03 | black merle | D/D | D/D | D/d3 | D/d3 | Dam of Mudi04-6,15,16 |
| Mudi | Mudi04 | grey merle | D/D | D/D | d3/d3 | d3/d3 | offspring |
| Mudi | Mudi05 | cream merle | D/D | D/D | d3/d3 | d3/d3 | offspring |
| Mudi | Mudi06 | grey | D/D | D/D | d3/d3 | d3/d3 | offspring |
| Mudi | Mudi15 | cream merle | D/D | D/D | D/d3 | D/d3 | offspring |
| Mudi | Mudi16 | black | D/D | D/D | D/d3 | D/d3 | offspring |
| Mudi | Mudi07 | black merle | D/D | D/D | D/d3 | D/d3 | Dam of Mudi08 |
| Mudi | Mudi08 | black merle | D/D | D/D | D/D | D/D | offspring |
| Mudi | Mudi09 | black | D/D | D/D | D/D | D/D |  |
| Mudi | Mudi10 | black | D/d1 | D/D | D/D | D/d1 |  |
| Mudi | Mudi11 | black | D/d1 | D/D | D/D | D/d1 |  |
| Mudi | Mudi12 | black | D/D | D/D | D/D | D/D |  |
| Mudi | Mudi13 | black | D/d1 | D/D | D/D | D/d1 |  |
| Mudi | Mudi14 | black | D/D | D/D | D/D | D/D |  |
| Mudi | Mudi01 | black merle | D/D | D/D | D/D | D/D |  |
| Mudi | Mudi17 | black merle | D/D | D/D | D/d3 | D/d3 |  |
| Mudi | Mudi18 | black merle | D/D | D/D | D/d3 | D/d3 |  |
| Mudi | Mudi19 | grey-brown merle | D/d1 | D/D | D/d3 | d1/d3 | offspring |
| Mudi | Mudi20 | black merle | D/d1 | D/D | D/d3 | d1/d3 | Dam of Mudi019 |
| Wolfdog | AW1 | black | D/d1 | D/D | D/D | D/d1 | Dam of AW2,3 |
| Wolfdog | AW2 | grey | d1/d1 | D/D | D/D | d1/d1 | offspring |
| Wolfdog | AW3 | grey | D/d1 | D/D | D/d3 | d1/d3 | offspring |
| Wolfdog | WD01 | grey fawn | D/D | D/D | d3/d3 | d3/d3 | Sire of WD04 |
| Wolfdog | WD04 | grey | D/D | D/D | d3/d3 | d3/d3 | offspring |
| Wolfdog | WD02 | grey | D/D | D/D | d3/d3 | d3/d3 |  |
| Wolfdog | WD03 | husky domino | D/D | D/D | D/D | D/D |  |
| Shih Tzu | ShT3 | grey | d1/d1 | D/D | d3/d3 | ? | Dam of ShT5,6 |
| Shih Tzu | ShT4 | white | D/D | D/D | D/D | D/D | Sire of ShT5,6 |
| Shih Tzu | ShT5 | not dilute | D/d1 | D/D | D/d3 | d1/d3 | offspring |
| Shih Tzu | ShT6 | not dilute | D/d1 | D/D | D/d3 | d1/d3 | offspring |
| Chihuahua | Ch1 | grey | D/d1 | D/D | D/d3 | d1/d3 |  |
| Italian Greyhound | IG1 | grey | D/d1 | D/D | D/d3 | d1/d3 |  |
| Pekingese | Pk1 | brown | D/D | D/D | D/d3 | D/d3 |  |
| Pekingese | Pk2 | white | D/d1 | D/D | D/d3 | d1/d3 |  |
